# Mfn2 Affects Embryo Development via Mitochondrial Dysfunction and Apoptosis

**DOI:** 10.1371/journal.pone.0125680

**Published:** 2015-05-15

**Authors:** Na Zhao, Yong Zhang, Qun Liu, Wenpei Xiang

**Affiliations:** 1 Family Planning Research Institute, Center of Reproductive Medicine, Tongji Medical College, Huazhong University of Science and Technology, Wuhan, Hubei, China; 2 Department of General Surgery, Union Hospital, Huazhong University of Science and Technology, Wuhan, Hubei, China; East Carolina University, UNITED STATES

## Abstract

**Background:**

Growth factors, energy sources, and mitochondrial function strongly affect embryo growth and development *in vitro*. The biological role and prospective significance of the mitofusin gene *Mfn2* in the development of preimplantation embryos remain poorly understood. Our goal is to profile the role of Mfn2 in mouse embryos and determine the underlying mechanism of Mfn2 function in embryo development.

**Methods:**

We transfected Mfn2-siRNA into 2-cell fertilized eggs and then examined the expression of *Mfn2*, the anti-apoptotic protein Bcl-2, and the apoptosis-promoting protein Bax by Western blot. Additionally, we determined the blastocyst formation rate and measured ATP levels, mtDNA levels, mitochondrial membrane potential (ΔΨm), and apoptosis in all of the embryos.

**Results:**

The results indicate that the Mfn2 and Bcl-2 levels were markedly decreased, whereas Bax levels were increased in the T group (embryos transfected with Mfn2-siRNA) compared with the C group (embryos transfected with control-siRNA). The blastocyst formation rate was significantly decreased in the T group. The ATP content and the relative amounts of mtDNA and cDNA in the T group were significantly reduced compared with the C group. In the T group, ΔΨm and Ca^2+ ^levels were reduced, and the number of apoptotic cells was increased.

**Conclusion:**

Low *in vitro* expression of *Mfn2* attenuates the blastocyst formation rate and cleavage speed in mouse zygotes and causes mitochondrial dysfunction, as confirmed by the ATP and mtDNA levels and mitochondrial membrane potential. Mfn2 deficiency induced apoptosis through the Bcl-2/Bax and Ca^2+^ pathways. These findings indicate that Mfn2 could affect preimplantation embryo development through mitochondrial function and cellular apoptosis.

## Introduction

Successful embryonic development requires a series of coordinated molecular and cellular events that culminate in blastocyst formation [1], and correct gene expression regulation is important in embryo development [2, 3]. The development of fertilized oocytes, including cleavage, blastocyst formation, and implantation, requires normal levels of specific gene expression and organelle activity [4]. Mitochondria occupy a key role in the management of many cellular functions, such as stress responses, cell metabolism, and cell death [5]. Mitochondria function as the energy source of cells, contribute to redox and Ca^2+^ homeostasis, provide intermediary metabolites, and store proapoptotic factors during embryonic development. In addition, mitochondria regulate Ca^2+^ homeostasis and modulate apoptosis through the release of a number of death inducing cell molecules [6, 7]. Moreover, mitochondria serve as a source of reactive oxygen species (ROS). Maintaining normal maternally derived mitochondrial function is critical for the early embryo. Mitochondrial dysfunction might disturb embryonic development and trigger apoptosis. This dual mitochondrial role might represent a control system that determines whether early embryo development proceeds normally or is quickly eliminated.

Mitofusin-2 (Mfn2) is a mitochondrial protein that controls mitochondria fusion and tethering [8, 9]; however, the latter study suggests that a small portion of Mfn2 is present in the endoplasmic reticulum (ER) [10, 11]. Mfn2, which is connected to altered mitochondrial energy supplies, is a signaling molecule that plays a vital role in cell activities [12]. Various studies have reported that Mfn2 plays a positive role in embryonic development [13–15]. If zygotes lack Mfn2, blastocyst formation is impaired [16]. However, the mechanism by which Mfn2 regulates embryo development remains unclear. The aim of this study was to further characterize the effects of Mfn2 and its mechanism of action during embryo development.

## Materials and Methods

The Ethics Committee of the Center of Reproductive Medicine of Tongji Medical College of Huazhong University of Science and Technology in China approved this study (permit number: 2011–149). All of the reagents used in the mouse fertilized-egg cultures were purchased from Sigma (St. Louis, MO, USA).

### Collection and Culture of Mouse Embryo Tissue Samples

The 5-week old Kunming white (KM) mice were primed with 10 IU of serum gonadotropin from pregnant mares (PMSG, The Bohn Pharmaceutical Co, Ltd., Chifeng, China) followed by 10 IU of human chorionic gonadotropin (hCG) 48 h later (Livzon Group Livzon Pharmaceutical Factory, Guangzhou, China) to obtain zygotes. The mice were mated with males of proven fertility, and plugs were verified the next morning. The 2-cell embryos were collected from oviduct at 48 h time point of post-hCG injections and cultured in M2 medium. The embryos were washed in IVF-30 thrice and transferred to 5% CO_2_-equilibrated IVF-30 (Vitrolife, Kungsbacka, Sweden). The embryos were cultured in a humidified incubator at 37°C and 5% CO_2_.

### siRNA-Mediated Mfn2 Knockdown

Dose dependent and time dependent experiments of siRNA transfected fertilized eggs were done and optimized methods was obtained. The 2-cell fertilized eggs were cultured in IVF-30 and transfected with siRNA (5 nM) for 6 h using Lipofectamine 2000 (Invitrogen) according to our optimized transfection methods. ON-TARGET plus siRNA-Mouse Mfn2 (T group) and non-targeting control siRNA (C group) were from Ruibo (Guangzhou, China). The transfection efficiencies were determined by qPCR. Fertilized eggs transfected were cultured and harvested for detecting at 4-cell (C1 and T1), 8-cell (C2 and T2) and blastocyst (C3 and T3) stages.

### RNA Isolation, Reverse Transcription, and PCR

Total RNA was extracted from 50 embryos using TRIzol according to the manufacturer’s instructions. Equal aliquots of total RNA from each group were quantified using a spectrophotometer at 260 nm and processed to synthesize the complimentary DNA (cDNA). cDNA was synthesized using 1 μg of RNA, oligo dT primers (Qiagen, Valencia, CA), and the Revert Aid First Strand cDNA Synthesis Kit (Fermentas, Rockford, USA) according to the manufacturer’s protocol. Amplification and SYBR Green II (Gene Copoeia, Maryland, USA) detection were performed using an MX3000P Real-Time PCR Detection System (Stratagene). The samples were run in triplicate, and the gene expression level for each sample was normalized to β-actin mRNA using the comparative threshold cycle (Ct) method.

### Western Blot

The protein samples (40 μg) were separated by SDS-PAGE and transferred to nitrocellulose membranes. Nonspecific binding was blocked with 0.01% TBS-Tween containing 5% nonfat milk for 1 h at room temperature. The membranes were hybridized with rabbit anti-Mfn2 (Abcam, Cambridge, MA), rabbit anti-Bax, rabbit anti-Bcl-2, and rabbit anti-β-actin primary antibodies (Santa Cruz Biotech, CA) in Tris-buffered saline with Tween (TBS-T) containing 1% nonfat milk at 4°C overnight. The membranes were washed three times with TBS-T, and horseradish peroxidase-conjugated secondary antibodies were used in a standard enhanced chemiluminescence reaction according to the manufacturer’s instructions (Pierce, Rockford, IL.).

### Determination of Mitochondrial Membrane Potential (ΔΨm)

Mitochondrial stability was assessed by fluorescence microscopy after incubation with JC-1 (5,59,6,69-tetrachloro-1,19,3,39-tetraethylben zimidazolylcarbocyanine iodide; Molecular Probes, Eugene, OR). The mouse embryos were grown in 35-mm Petri dishes in 40 μl of IVF-30. The fertilized eggs were incubated with 0.1 μM JC-1 (Cayman Chemical Company, USA) fluorescent dye for 30 min in the CO_2_ incubator and then slowly washed with PBS several times. The mitochondrial membrane potential was evaluated under a fluorescence microscope using a 540/570 nm filter. The green JC-1 signals were measured at 485/535 nm, whereas the red signals were measured at 590/610 nm.

### Detection of Apoptosis

The embryos were analyzed for apoptosis with annexin V-FITC using a fluorescence microscope. The blastocysts were fixed to allow for annexin V-FITC labeling of cells with an intact plasma membrane, whereas fertilized eggs with disrupted or otherwise damaged plasma membranes were stained with propidium iodide (PI). Following three washes with PBS at 37°C, the blastocysts were incubated in a binding buffer that contained 1 μl/100 μl annexin V-FITC and 1 μl/100 μl PI in a Petri dish for 10 min at room temperature while avoiding light exposure. After the incubation, the fertilized eggs were washed in PBS, and then immediately observed under an inverted fluorescence microscope with the appropriate bandwidth filters for green and red fluorescence.

### Determination of ATP Levels

To evaluate mitochondrial function, we determined ATP levels in the embryos. The reagent was dissolved in PBS on ice, and the ATP standard solution (The Shenyang Branch Liang Horse Biological Engineering Co, Ltd., China) was diluted with double distilled water to a suitable concentration gradient. Thirty blastocysts from each group were collected in PBS. After three washes, the blastocysts were suspended in calcium and magnesium ion-free PBS. Then, 100 μl of ATP-detection working solution was added to each well of a 96-well plate, and a standard curve was plotted using multifunctional microplate fluorescence emission values. We added ATP detection lysates and ATP detection reagent to each of the detection holes and measured the luminescence using an automatic microplate reader (BIO-TEK).

### Detection of Free Cytoplasmic Ca^2+^

Fluo-3 imaging reveals spatial dynamics in Ca^2+^ signaling, and fluo-3 absorbs the compatible spectrum with excitation at 488 nm by argon ion laser sources. A significant increase in fluorescence intensity in response to Ca^2+^ binding is observed. The zygotes were washed three times in PBS without Ca^2+^ and then incubated in fluo-3 solution (1 mM) for 15–30 min at 37°C and 5% CO_2_. After an additional PBS wash, the zygotes were observed under a fluorescence microscope.

### Quantification of mtDNA Relative to Nuclear DNA (Mt/N)

One hundred blastocysts from each group were collected to prepare total DNA using the DNeasy Blood & Tissue Kit (Qiagen, Germany). The total DNA was dissolved in 10 μl of solution, and the mtDNA content was assessed by quantification of *CoxII* relative to the nuclear gene *β-actin*. The CoxII primer sequences were as follows: forward 5′-GAGCAGTCCCCTCCCTAGGA-3′ and reverse 5′-GTCG GTTTGATGTTACTGTTGCTT-3′. Nuclear and mitochondrial DNA contents were quantified by real-time PCR. For each gene, PCR quantification was performed in triplicate, and the amplified transcripts were quantified using the comparative Ct method. Briefly, the Ct values were calculated according to the following equation: ΔCt = Ct_CoxII_—Ct_β-actin_, where ΔCt is the difference in the Ct values between CoxII and β-actin. The relative quantity of DNA expression in the silent gene groups was compared with the control groups. Xn is calculated by the formula Xn = 2^-ΔCt^.

### Statistical Analysis

The data were analyzed using the Statistical Program for Social Science (SPSS, Inc., Chicago, IL, USA) software. Each experiment was performed at least three separate times, and the data were presented as the mean ± standard error (x±s). Significance was established at the 95% confidence level (P<0.05).

## Results

### Mfn2 Expression in Fertilized Mouse Eggs after Transfection

**Fig 1:**
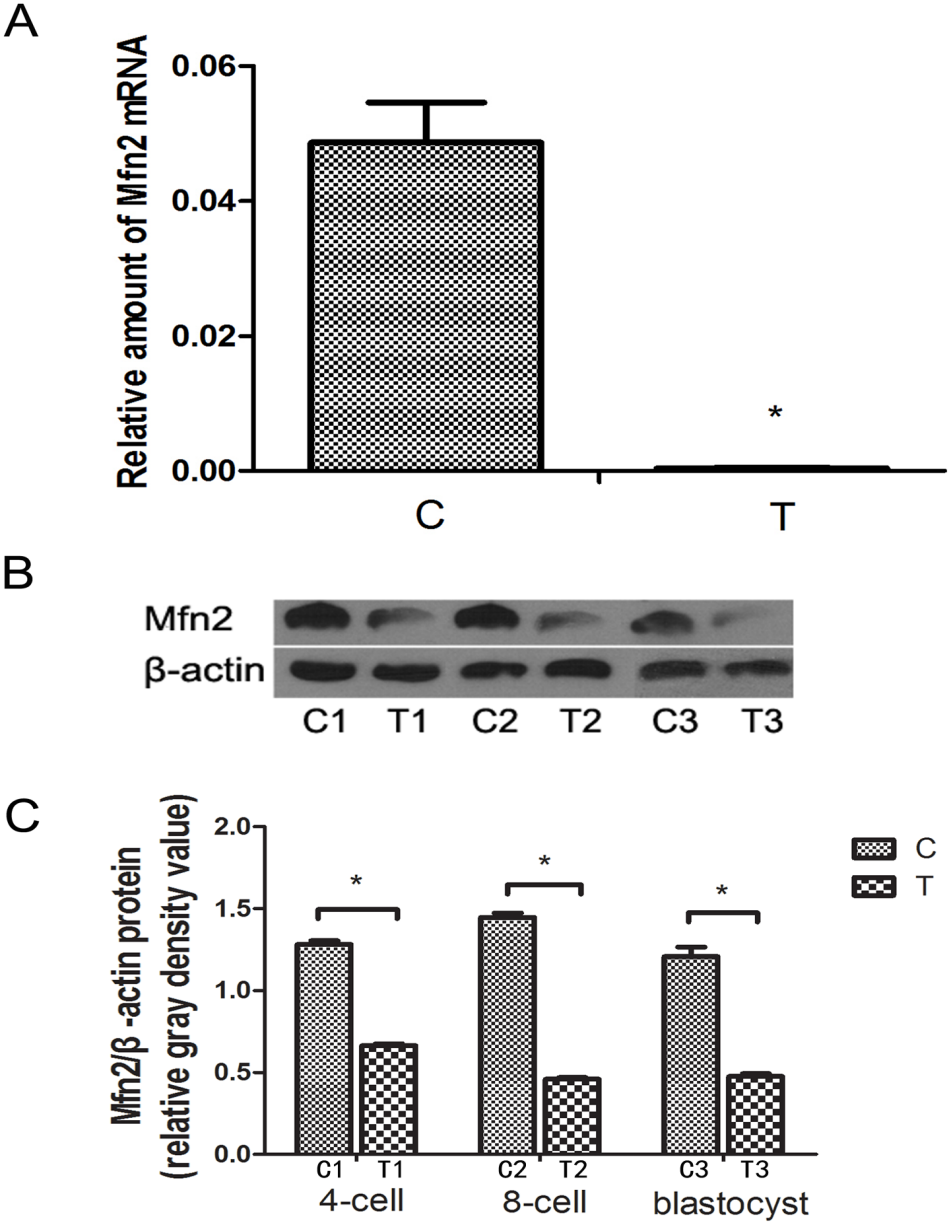
Mfn2 expression in mouse fertilized eggs after transfection. Mfn2 mRNA expression was detected by qPCR and its level is significant lower in T group than that in C group (A). Mfn2 protein levels were determined by Western blotting (B). The quantitation of Mfn2 protein levels. C1 and T1: 4 cells fertilized eggs, C2 and T2: 8 cells fertilized eggs, C3 and T3: blastocyst. *P<0.05. Data are means ± SD of 3 separate experiments (C).

We measured *Mfn2* expression by PCR and Western blotting and found that the mRNA levels were decreased in the fertilized eggs treated with Mfn2-siRNA compared with eggs transfected with the control-siRNA (Fig 1A). Western blot analysis confirmed these findings (Fig 1B). At the 4-cell stage, the *Mfn2* expression level was significantly reduced, and this low expression continued until the blastocyst stage (Fig 1C).

### Low *Mfn2* Expression Attenuates the Blastocyst Formation Rate and Cleavage Speed

**Fig 2:**
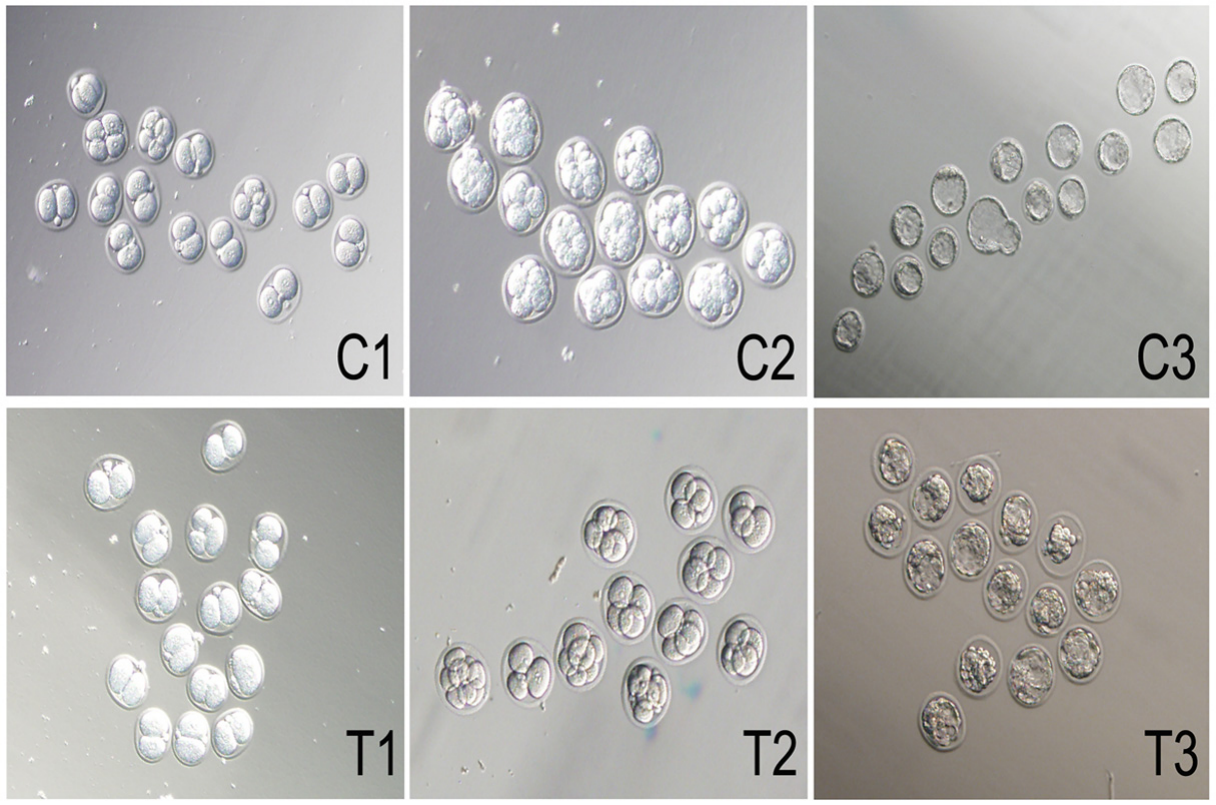
Low Mfn2 expression attenuates the blastocyst formation rate and cleavage speed. In T group, the cleavage speed was reduced compared with that in control group, the blastocyst formation rate was significantly reduced in the T group compared with the C group. C1 and T1: 4-cell fertilized eggs, C2 and T2: 8-cell fertilized eggs. C3 and T3: Blastocyst. Data are means ± SD of 3 separate experiments.

To examine the effects of Mfn2 on embryo development, we determined the blastocyst formation rate and cleavage speed of the embryos in two groups. The blastocyst formation rate was significantly reduced in the T group (embryos transfected with Mfn2-siRNA) compared with the C group (embryos transfected with control-siRNA). When the 2-cell fertilized eggs were removed at 72 h, 72.95% blastocyst formation was noted in the C group, whereas 23.19% was observed in the T group (p<0.01, Table 1). In the T group, the cleavage speed was reduced compared with the control group. After 24 h, the 2-cell fertilized eggs were removed and examined. Approximately 80% of the zygotes developed into the 4-cell stage (Fig 2C1 and 2T1). At 36 h, approximately 80% of the cells entered the 8-cell stage in the C group (Fig 2C2), whereas only a limited number had entered this stage in the T group (Fig 2T2). After 72 h, 80% of the cells were blastocysts in the C group (Fig 2C3); the cells in the *Mfn2*-deficient group were dead (Fig 2T3). These results suggest that normal *Mfn2* expression is essential for the development of preimplantation embryos.

**Table 1:** Blastocyst formation rate.

Groups	Control-siRNA transfected	Mfn2-siRNA transfected
The number of 2-cell fertilized eggs	122	138*
The number of developed blastocysts	89	32**
Blastocyst formation rate (%)	72.95	23.19**

The blastocyst formation rate of the fertilized eggs collected from 5-week-old Kunming mice. The table presents the number of fertilized eggs collected from five mice. The values represent the rate of successful blastocyst development.

* *P*>0.05 *vs* control-siRNA transfected,

** *P*<0.01 *vs* control-siRNA transfected.

### Low *Mfn2* Expression causes Mitochondrial Dysfunction in Mouse Embryos

**Fig 3:**
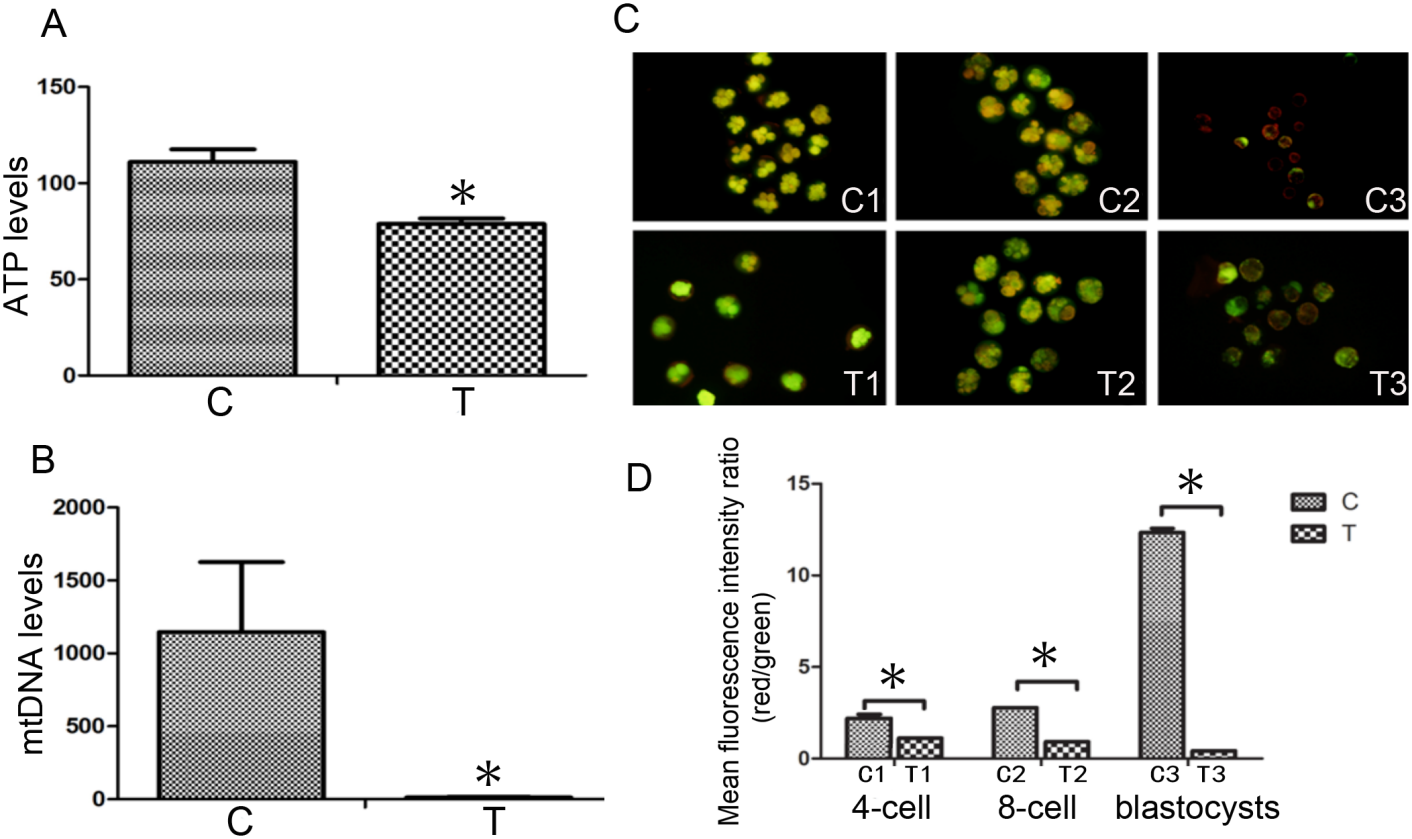
A) The ATP content of the mouse blastocysts was detected via firefly luciferase analysis. B) The relative expression levels of mtDNA and cDNA. Values are presented as multiples of COXII DNA relative to the β-actin DNA. C) Mitochondrial membrane potential was assessed by JC-1. D) Statistical analyses of the red and green mean fluorescence intensity ratios revealed that the mitochondrial membrane potential of the T group was significantly reduced compared with the C group, *P<0.05. Data are means ± SD of 3 separate experiments.

To further determine the possible effects of Mfn2 on embryo development via mitochondrial function, we measured ATP levels and mtDNA production in fertilized eggs treated with control-siRNA or Mfn2-siRNA. The results revealed that the ATP content of the 30 mouse blastocysts in the T group was significantly reduced compared with the C group (*P*<0.05; Fig 3A). COXII and β-actin served as markers for mitochondrial DNA (mtDNA) and nuclear DNA (cDNA), respectively. The relative ratio of mtDNA to cDNA (Fig 3B) was determined showing reduction in the T group compared with the C group.

We assessed ΔΨm using the JC-1. At the 4-cell (Fig 3C, 3C1 and 3T1) and 8-cell stages (Fig 3C, 3C2 and 3T2), ΔΨm levels were low in the C and T-groups. However, a difference in these levels was noted between the two groups; ΔΨm was significantly increased (red) in the C group compared with the T group (green; Fig 3C, 3C3 and 3T3; Fig 3D). These results suggest that *Mfn2* participates in early mouse embryonic development by affecting mitochondrial function through energy metabolism and the quantity of mtDNA.

### Low *Mfn2* Expression Induces Apoptosis in Mouse Embryos

**Fig 4:**
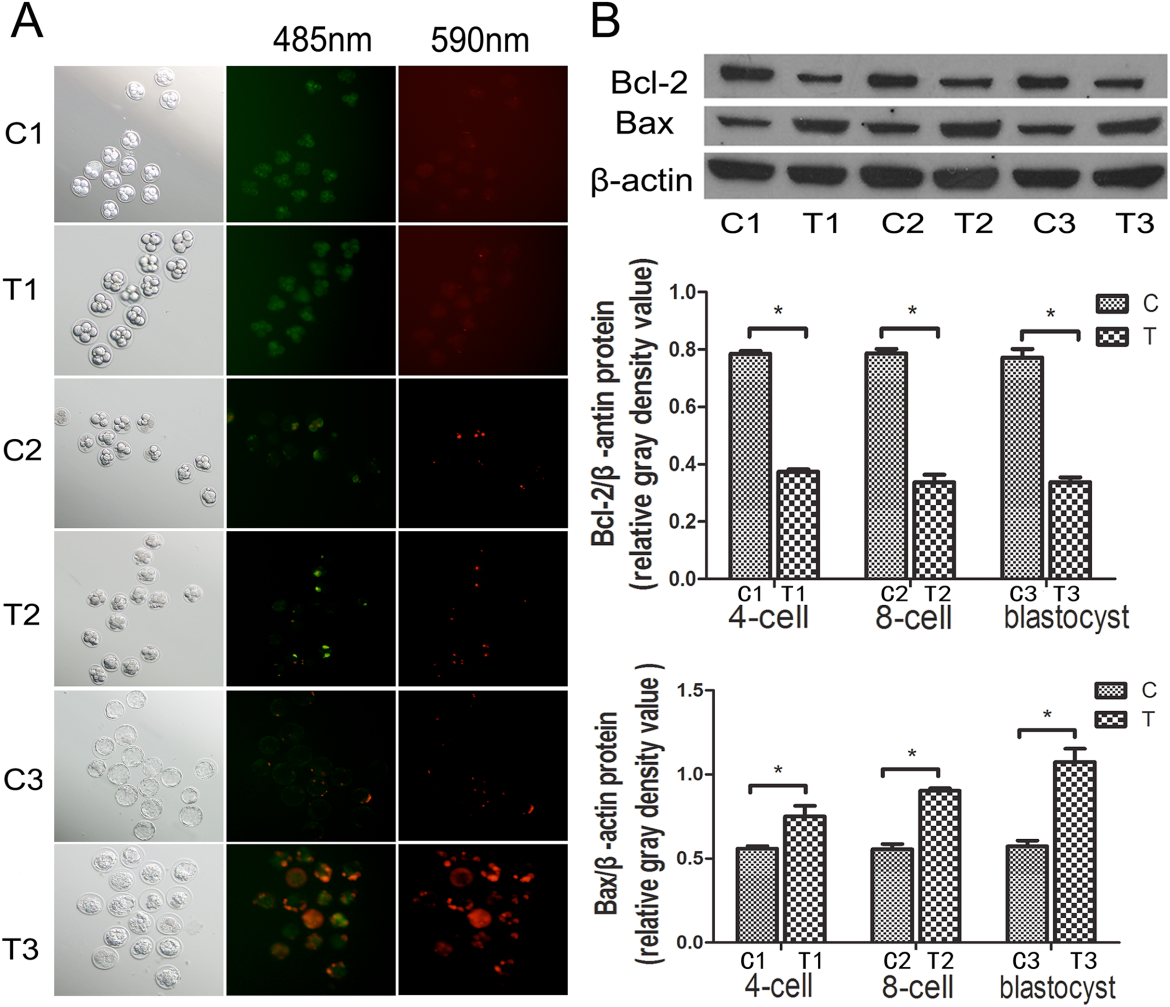
A) Annexin V and PI stains were observed at 485 nm and 590 nm, respectively, using a fluorescence microscope and were used to visualize the cells undergoing apoptosis (green) and death (red), respectively. B) Bcl-2 and Bax protein levels were determined by Western blot and quantitation was showed that Bax expression increased significantly and Bcl-2 expression decreased in the T group compared with the C group. *P<0.05. Data are means ± SD of 3 separate experiments.

The fluorescence microscope images of the annexin V and PI staining indicated that only a small number of cells at the 4-cell stage in the two groups were undergoing apoptosis or death. At the 8-cell stage, few apoptotic or dead cells were observed in the T group (Fig 4AT2), and evidence of these cellular processes were not obvious in the C group (Fig 4AC2). At the blastocyst stage, the number or apoptotic and dead cells in the T group (Fig 4AT3) increased significantly (Fig 4AC3). We verified the occurrence of apoptosis through detection of the apoptosis-related proteins Bcl-2 and Bax by Western blot (Fig 4B). Using a gray scale analysis, we discovered that Bax expression increased gradually in the T group, whereas overall Bcl-2 expression decreased. A statistically significant difference in the gray value was observed in the 8-cell and the blastocyst stages (Fig 4B). These results indicate that low *Mfn2* expression potentially leads to the development of a disordered preimplantation embryo by promoting apoptosis.

### Low *Mfn2* Expression causes Decreased Cytoplasmic Ca^2+^ Levels

**Fig 5:**
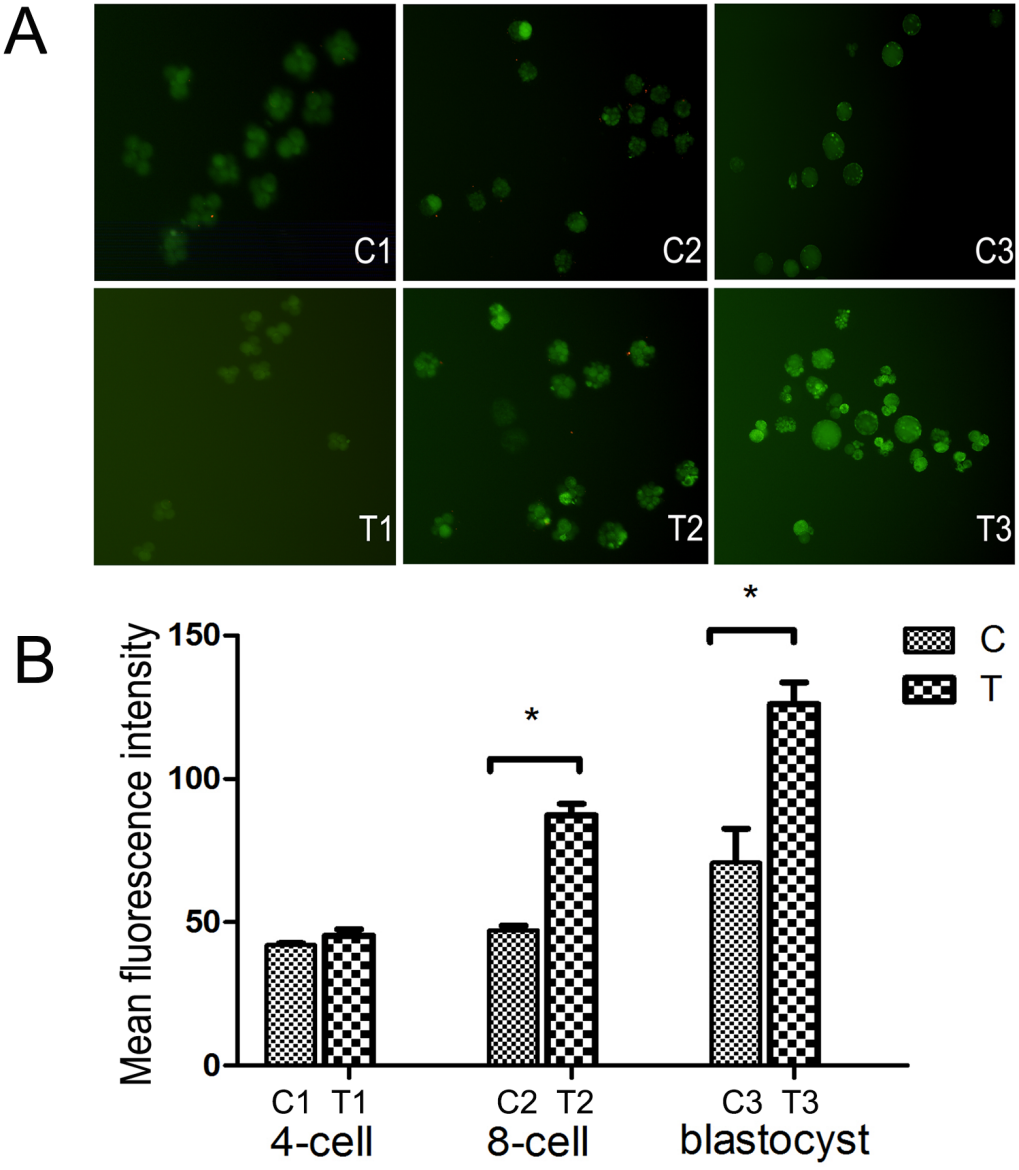
A) Fluo-3 colored fluorescence to measure the Ca^2+^ levels. B) The Ca^2+^ level difference was shown between the C and T-groups in the fertilized eggs and blastocysts. *P<0.05. Data are means ± SD of 3 separate experiments.

We used fluo-3 colored fluorescence to measure the Ca^2+^ levels. The free Ca^2+^ level gradually increased in the T group (Fig 5) during the observation period, whereas no obvious change was noted in the C group. The fluorescence intensity analysis indicated a statistically significant difference between the groups.

## Discussion

In this study, we observed that low *Mfn2* expression affects the development of mouse preimplantation embryos by obstructing mitochondrial metabolism. Mitochondria, which are maternally inherited, have a well-established role in determining embryo quality [14], ΔΨm, ROS, and metabolic outputs. Mitochondrial content is an important indicator of cellular health, reflecting overall mitochondrial integrity and metabolic activity [17–19].

Our results indicate that ATP levels were significantly reduced in the *Mfn2*-deficient group compared with the control group, indicating that low *Mfn2* expression potentially causes mitochondrial dysfunction. Mfn2 plays a central role in mitochondrial outer-membrane fusion and mitochondrial function regulation. The process of preimplantation embryo development requires large amounts of ATP to provide the necessary energy [20, 21]. *Mfn2*-deficiency resulted in a reduced rate of ATP production and did not provide sufficient energy for embryonic cell division. This effect could cause the reduced division speed in the T group compared with the C group.

In addition, ΔΨm is an indicator of mitochondrial function, which reflects the metabolic activity of the mitochondrial membrane [4]. Our results indicate that the ΔΨm in the *Mfn2*-deficient group was considerably reduced compared with the control-siRNA transfected group, indicating that *Mfn2* deficiency likely results in mitochondrial dysfunction. Previous studies have demonstrated that pre-compaction stage embryos exhibit reduced levels of cellular activities, including low respiratory rates, O_2_ consumption, glucose utilization [22–27], and ΔΨm levels. As the embryo divides, the metabolic parameters change, and the metabolic activity of the reduced number of mitochondria per cell must increase to meet the increasing demands of cellular activity [28, 29], and the ΔΨm remains at a high level. Many mitochondrial functions, including ATP generation, Ca^2+^ homeostasis, and protein import, depend on the maintenance of ΔΨm [30]. Our results indicated that Mfn2 deficiency results in a low ΔΨm and failure to meet the demands of cellular activity, leading to disordered embryonic development. This finding demonstrates that normal Mfn2 expression is necessary to support metabolic activity.

Previous studies have demonstrated that mtDNA levels remain low after fertilization; however, levels begin to increase near the time of implantation [31]. The expression of mtDNA is upregulated, causing the reactivation of mtDNA replication at the blastocyst stage. Mitochondria begin to differentiate into elongated organelles with increased membrane potential and oxygen consumption; mitochondria exhibit increased oxidative phosphorylation activity, which is important for ATP production [32]. In our study, we observed that the mtDNA/cDNA ratio was increased at the blastocyst stage in the control-siRNA transfected group compared with the Mfn2-siRNA transfected group. This effect might contribute to the developmental disorders in Mfn2-siRNA transfected blastocysts.

Another possible mechanism involves the role of Mfn2 in the regulation of apoptosis; Mfn2 expression has an apoptotic effect in the mitochondrial apoptotic pathway [13]. Bcl-2 is a member of the human proto-oncogene Bcl-2 family [33], which includes pro-apoptotic and anti-apoptotic proteins [34]. Bax does not induce apoptosis until it is translocated to the mitochondria [35]. Bax and Bcl-2 activation prompts cytochrome c translocation into the cytoplasm via the outer mitochondrial membrane, which leads to the activation of the caspase cascade and subsequent apoptosis [36, 37]. In this study, Mfn2 deficiency profoundly increases the mitochondrial Bax/Bcl-2 ratio, indicating that Mfn2 deficiency potentially induces apoptosis in the mouse embryo through the Bcl-2 and Bax pathway.

In addition to apoptotic cells, we also observed numerous dead cells in the Mfn2 siRNA-transfected mouse embryos. The death of these cells potentially occurred through programmed necrosis, which is dominated by Ca^2+^-dependent permeabilization of the outer and inner mitochondrial membranes. Previous studies have demonstrated that mitochondria are central regulators of programmed cell death, apoptosis, and necrosis [38] that Ca^2+^ transported from the sarcoplasmic reticulum (SR) to mitochondria is dependent upon “Ca^2+^ hot-spots”. Because Mfn2 is an important protein that creates Ca^2+^ hot spots by tethering to the organelle, Mfn2 deficiency could promote cell programming. In our results, Mfn2 downregulation in mouse embryos caused increased Ca^2+^ concentrations and cell mortality and reduced energy metabolism; this finding suggests that a defect in Mfn2 expression could induce abnormal Ca^2+^ transportation, which is potentially involved in apoptosis.

Our results demonstrate that inadequate *Mfn2* expression affects embryonic development and that one of the mechanisms involves mitochondrial dysfunction and apoptosis induced by low *Mfn2* expression. These findings indicate that Mfn2 plays a decisive role in successful preimplantation development. Further investigation is needed to address the mechanism of Mfn2 regulation signaling.

## Supporting Information

S1 File Formatting summary and additional instructions. This figure is a certification that American Journal Experts (AJE) has edited our manuscript, including citations and references and AJE reminded us to pay attention the additional instructions.
(PDF)

S2 File Editorial certificate. This figure shows that our manuscript has been edited by American Journal Experts and AJE has changed our manuscript to meet PLOS guidelines and provided language editing, translation, manuscript formatting, and figure formatting to ensure our manuscript meets submission guidelines.
(PDF)
