# Supplementary material for: Mfn2 Affects Embryo Development via Mitochondrial Dysfunction and Apoptosis
Source: PLoS One. 2015 May 15;10(5):e0125680. doi: 10.1371/journal.pone.0125680 (PMC4433325; doi:10.1371/journal.pone.0125680)
Supplement: S2 File — This figure shows that our manuscript has been edited by American Journal Experts and AJE has changed our manuscript to meet PLOS guidelines and provided language editing, translation, manuscript formatting, and figure formatting to ensure our manuscript meets submission guidelines. (PDF) [file pone.0125680.s002.pdf]

Client: Professor wenpei xiang

Manuscript: Mfn2 affects embryo development via mitochondrial dysfunction and apoptosis

Journal: PLOS ONE

Formatting Summary and Additional Instructions

Prepared: November 17, 2014

Congratulations—you're one step closer to submission!
This report summarizes the work that has been done to bring your manuscript into compliance with your targeted journal's formatting requirements, and it describes any further changes that you will need to make before submitting your manuscript to your specified journal. Please review this document and the marginal comments in your manuscript carefully. If you have any questions, please contact us at support@aje.com.

Citations and References

Your citations and reference list were corrected for spelling, author lists, journal abbreviations, and format according to the journal's specified style.

Figures and Figure Legends

Per the journal guidelines, each figure should be submitted as a separate file. If you would like assistance preparing figures, please contact support@aje.com to use our Figure Formatting service.
